# Editorial: HSPs—Ambiguous Mediators of Immunity

**DOI:** 10.3389/fimmu.2016.00639

**Published:** 2016-12-26

**Authors:** Stuart K. Calderwood, Ayesha Murshid, Thiago J. Borges

**Affiliations:** ^1^Beth Israel Deaconess Medical Center, Harvard Medical School, Boston, MA, USA; ^2^Pontifícia Universidade Católica do Rio Grande do Sul (PUCRS), Porto Alegre, RS, Brazil

**Keywords:** heat shock protein, immunity, vaccine, mycobacterial Hsp65, transplant, arthritis

**Editorial on the Research Topic**

**HSPs—Ambiguous Mediators of Immunity**

Heat shock proteins (HSPs) were discovered as polypeptides induced by stress that can be found in all kingdoms of cellular organisms. Their functions were, a first enigmatic and these proteins were thus classified by molecular weight, as in—Hsp27, Hsp70, Hsp90, Hsp110 (1). More recently, each of these size-classified molecules has attributed a role in protein folding, and they thus came to be known, as a class, as molecular chaperones—deterrents of unsuitable interactions between intracellular proteins (2). However, the HSPs possess properties beyond chaperoning. Indeed, their discovery in the extracellular spaces suggested roles in intercellular signaling and in the convoluted regulation of the immune responses.

A number of lines of investigation triggered interest in HSPs as mediators of immunity. Srivastava and others found that the molecular chaperone properties of HSPs could be harnessed in cancer vaccine design (3–5). They emphasized the role of HSPs in capturing tumor antigens and permitting their uptake and processing by antigen-presenting cells (APCs) prior to activation of cytotoxic lymphocytes. Others suggested that HSPs could behave like endogenous danger signals when flooding into the extracellular microenvironment after cell death (6). In another line of investigation, investigators studied the role of mycobacterial Hsp65 and Hsp70 in suppression of autoimmune diseases such as arthritis, diabetes, and prolongation of tissue grafts [Borges et al.; (7, 8)]. HSPs were thus implicated in contrasting and apparently opposed aspects of immunity.

The current volume contains articles dealing with these aspects of HSP biology. Four articles describe various roles of HSPs in tumor immunity and anticancer vaccine construction. In chapter 1, Zuo et al. describe tumor immunity strategies built around the “large HSPs”—Hsp110 and GRP170. These larger HSPs possess chaperoning power of remarkable strength leading to high avidity for antigens and effective vaccines. Shevtsov and Multhoff in chapter 4 review in detail Hsp70 and Hsp90 vaccines and their effectiveness in tumor therapy. The biological properties of Hsp90 are further discussed in chapter 5 by Tamura et al., emphasizing the role of this molecule in permitting antigens to cross plasma membranes, enter cells by endocytosis and cross the endosomal wall to the sites of antigen processing and acquisition by MHC Class I molecules. Most studies have indicated a role for surface receptors in mediating effects of extracellular HSPs. Murshid et al. in chapter 7 stress the role of scavenger receptors in the immune functions of such HSPs, concentrating on SRECI/*SCARF1* as an avid binder of most of the HSPs.

Four additional chapters concentrate on the immunoregulatory properties of HSPs, particularly emphasizing study of mycobacterial chaperones. In chapter 2, Manon et al. concentrate on mycobacterial Hsp70 and describe the generation of the first TCR transgenic mouse recognizing an anti-inflammatory Treg (regulatory T cell)-inducing Hsp70 peptides. The aim was to provide a model system for discovery of the mechanisms underlying generation of Hsp70-reactive CD4^+^CD25^+^ Treg and mediation of immunomodulation. In chapter 6, Moudgil et al. describe their interesting studies of the role of mycobacterial Hsp65 in controlling adjuvant arthritis in rodent models and potential development of novel treatments based on these findings. Along similar lines Borges et al. describe studies showing a potent role for mycobacterial HSPs in regulating alloimmunity and improving survival of tissue grafts. Finally, the O’Brien group (chapter 8) describes roles for the small HSP—Hsp27 in attenuating atherogenesis and other events in the extracellular spaces and in the circulation.

Finally, Calderwood et al. in chapter 3 attempt to synthesize some of theses apparently contrasting immunostimulatory and immunoregulatory effects of HSPs and develop an integrated understanding of potential *sequela* of HSPs encountering macrophages or dendritic cells.

Thus, from the analysis contained in this collection of articles, we appear to have come a long way in past 30 years in understanding “the other face of HSP biology”—the various families of HSPs escaping to the extracellular milieu and influencing immunity. However, much remains to be learned in terms of the recognition of HSPs by receptors on APC, in cell signaling and in understanding how these events are influenced by tissue context. In addition, the complex pathways undertaken by HSP-chaperoned peptides in the intracellular milieu and their influence on antigen presentation remain to be fully characterized. In terms of translation of HSP research to disease treatment, promising approaches to cancer immunotherapy, treatment of inflammatory disease such as arthritis and survival of tissue transplants appear to beckon. This area of HSP biology thus appears to have rich promise for the future.

## Author Contributions

SC, AM, and TB each contributed equally to writing this editorial.

## Conflict of Interest Statement

The authors declare that the research was conducted in the absence of any commercial or financial relationships that could be construed as a potential conflict of interest.
